# An effect of positional isomerism of benzoic acid derivatives on antibacterial activity against *Escherichia coli*

**DOI:** 10.1515/biol-2021-0060

**Published:** 2021-06-19

**Authors:** Alicja Synowiec, Kinga Żyła, Małgorzata Gniewosz, Marek Kieliszek

**Affiliations:** Department of Food Biotechnology and Microbiology, Institute of Food Sciences, Warsaw University of Life Sciences–SGGW, Nowoursynowska 159C, 02-776 Warsaw, Poland

**Keywords:** benzoic acid derivatives, positional isomerism, antibacterial activity, *E. coli*

## Abstract

This study demonstrated the effect of positional isomerism of benzoic acid derivatives against *E. coli* ATCC 700728 with the serotype O157. The addition of hydroxyl and methoxyl substituents weakened the effect of acids against *E. coli* with respect to benzoic acid (except 2-hydroxybenzoic). The connection of the hydroxyl group at the second carbon atom in the benzoic ring reduced the time needed to kill bacterial cells. Phenolic acids with methoxyl substitutes limited the biofilm formation by *E. coli* to a greater extent than hydroxyl derivatives. The most significant influence on the antibacterial activity of phenolic acids has the type of substituent attached to the benzoic ring, their number, and finally the number of carbon atoms at which the functional group is located.

## 1 Introduction

A growing interest in natural compounds and their potential use in the food industry has been observed in recent years [1]. Benzoic acid and its salts are widely used as food preservatives. This is due to the lack of color, low price, easy inclusion in a food product, and a wide spectrum of antimicrobial activity [2]. Some derivatives of weak acid were more effective than the parent acid [3]. The group of such compounds includes phenolic acids, which are widespread in nature as secondary plant metabolites. Studies demonstrated that phenolic acids inhibit the development of many pathogenic microorganisms such as *Staphylococcus aureus*, *Escherichia coli*, *Klebsiella pneumoniae*, *Bacillus cereus*, *Aspergillus flavus*, and *Aspergillus parasiticus* [1]. Phenolic acids are organic compounds, containing an aromatic ring, hydroxyl or methoxyl substituent, and a carboxyl group in their chemical structure. In various plant extracts, benzoic acid derivatives differ in number, location, and type of substituent at the benzoic ring. Hydroxyl derivatives of benzoic acid may include salicylic, protocatechuic, or gallic acid, while methoxyl derivatives include *p*-anisic and veratric acid. Few scientific studies indicate that the antimicrobial action of phenolic compounds is related to the presence of hydroxyl or methoxyl groups in the acid molecule. The available literature also reports that the inhibitory effect against bacteria is related to the number, type, and position of substituents at the benzene ring [4,5]. The antimicrobial activity of phenolic acids consists of lowering the pH of the environment, which directly inhibits the growth of bacterial cells. Studies demonstrated that any form of acid could potentially become incorporated into or penetrate the structure of the phospholipid layer, acidifying the intracellular pH and/or interacting with cellular components. The incorporation of phenolic acid molecules into the phospholipid layer of the bacterial membrane disrupts van der Waals interactions between the acyl lipid chains, leading to membrane disintegration. As a result, the ion gradient is disturbed, leading to the release of essential components from the bacterial cell and its death [6]. The structure of the bacterial cell influences the antimicrobial action of phenolic acids. The presence of lipid envelope in Gram-negative bacteria gives the cell surface a more nonpolar character, hindering the entry of polar particles into the cytosol [7]. The aim of the study is to analyze the effect of the number of substituents and positional isomerism in benzoic acid derivatives on the antibacterial activity against *E. coli*.

## 2 Materials and methods

### 2.1 Biological material and phenolic acids

The test bacterium was *Escherichia coli* ATCC 700728 with the serotype O157. The phenolic acids were purchased from Sigma-Aldrich (St. Louis, USA). Basic acid solutions were prepared in 96% ethanol (Avantor, Poland), according to Table 1.

**Table 1:** Phenolic acids tested

Phenolic acids	Structural formula	Acid solutions (%m/v)	log *P* (PubChem)	p*K*_a_ (PubChem)
Benzoic acid (Ba)	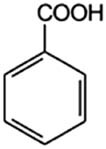	10	1.9	4.19
2-Hydroxybenzoic acid (2hBa)	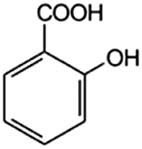	10	2.3	2.97
3-Hydroxybenzoic acid (3hBa)	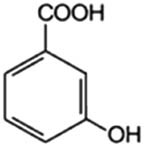	10	1.5	—
4-Hydroxybenzoic acid (4hBa)	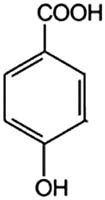	10	1.6	4.54
3,4-Dihydroxybenzoic acid (3,4hBa)	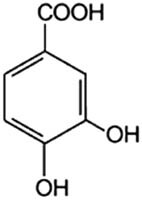	5	1.1	4.26
3,4,5-Trihydroxybenzoic acid (3,4,5hBa)	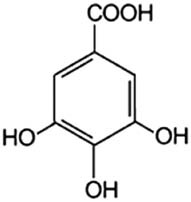	10	0.7	4.40
2-Methoxybenzoic acid (2mBa)	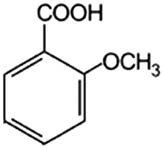	5	1.6	—
3-Methoxybenzoic acid (3mBa)	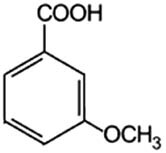	5	2.0	—
4-Methoxybenzoic acid (4mBa)	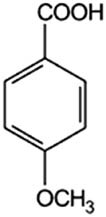	5	2.0	4.47
3,4-Dimethoxybenzoic acid (3,4mBa)	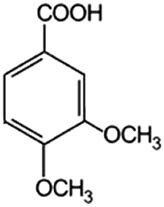	5	1.6	—

–, no data; log *P*, partition coefficient between octanol and water; p*K*_a_, acid dissociation constant.

### 2.2 Determination of minimum inhibitory concentration (MIC)

To determine the MIC of phenolic acids, we used a serial microdilution method according to the Clinical Laboratory Standards Institute [8]. Two series of dilutions of the examined acids were prepared in Müller-Hinton broth (MHB, Merck, Germany) in the concentration range of 0.13–8.0 mg/mL using 96-well plates. Inoculum of *E. coli* was added to each well of the plate containing 250 µL (MHB including acid), so that the final number was 5 × 10^5^ cfu/mL. The plates with bacteria were incubated at 37°C for 20 h. After 20 h, 25 µL of resazurin at 0.02% m/v (filtered through a 0.22 µm sterile filter) was added to each well. The plates were then incubated at 37°C for 2 h. Bacterial growth was assessed based on the change in resazurin color from violet to pink [9]. The MIC value was defined as the lowest concentration of phenolic acid in which no growth of *E. coli* was observed (no change in resurine color). The medium containing MHB together with the inoculum was used as a positive control. The pH values of the media containing acids were checked. The minimum pH value at which *E. coli* bacteria growth is inhibited was determined (pH 3.9).

### 2.3 Determination of minimum bactericidal concentration (MBC)

The determination of MBC was determined using a series of steps, undertaken after a MIC test has been completed. The test consisted of plating a 0.1 mL culture of the medium from the well, in which no bacterial growth was observed (during the MIC test) on Petri dishes with Muller-Hinton (MHA) agar. Incubation was carried out for 24 h at 37°C. Then, the number of grown bacterial colonies was calculated. MBC is defined as the lowest concentration, which causes 99.9% reduction in the number of living bacteria cells [10].

### 2.4 Time kill study

Liquid cultures in MHB (Merck, Germany) containing phenolic acids at concentrations corresponding to MIC and three times higher (3MIC) containing 10^5^ cfu/mL *E. coli* were incubated at 37°C for 24 h. During culture, the number of live bacteria was determined at 0, 60, 120, 240 min and 24 h by inoculating 20 µL from appropriate dilutions on MHA medium (BTL, Poland) and incubating at 37°C for 24 h. The first time when no live bacteria were found was taken as a result. The determination was performed in three repetitions [11]. The pH values of the media containing acids were checked.

### 2.5 Biofilms formation

The formation of biofilms was studied using the method described by Al-Shabib et al. [12] with modification. Phenolic acids at concentrations from 1 MIC to 1/16 MIC were added to Luria-Bertani (LB) broth (Oxoid, USA). *E. coli* inoculum was added to the medium, so that their final concentration was 5 × 10^5^  cfu/mL. Then, it was transferred to three wells in a 100-well flat-bottomed polystyrene plate (Honeycomb, flat-bottomed) and incubated at 37°C for 48 h. The plates were then gently washed three times with phosphate-buffered saline solution (PBS; pH 7.4) and stained with 100 mL 0.1 m/v crystal violet (Sigma-Aldrich, USA) for 30 min at room temperature (25°C). Excess crystal violet was removed by washing three times with PBS solution and suspended again in 150 µL 96% ethanol. The biofilm was quantified by optical density (OD) measurement at *λ* = 600 nm. The tests were carried out in three repetitions. The negative control was LB broth, and the positive control was LB broth with *E. coli*. The difference between the OD_600_ value of LB medium and the sample containing phenolic acids or control was used to assess the biofilm-forming ability of *E. coli*. The amount of biofilm formed was evaluated according to the formula (1):

(1) {B}_{\text{f}}=({\text{OD}}_{\text{a}}\times 100\text{\%})/{\text{OD}}_{\text{c}},]

where *B*_f_ is the amount of biofilm formed with respect to the control (%), OD_a_ is the OD_600_ value of LB medium containing phenolic acid, and OD_c_ is the OD_600_ value of LB medium with *E. coli* (positive control).

The results were interpreted according to the following scale:

*B*_f_ > 100 the examined acid stimulates *E. coli* to produce a biofilm.

*B*_f_ = 100 the examined acid does not affect the amount of biofilm produced by *E. coli.*

*B*_f_ < 100 the examined acid reduces the amount of biofilm produced by *E. coli.*

### 2.6 Statistical analysis

A statistical analysis was carried out for all the results obtained, including the calculation of the arithmetic mean. A normality test for OD biofilms was performed using the Shapiro–Wilk test and a homogeneity test of variance using the Levene or Brown–Forsythe tests. The significance of differences between the mean values was verified using an analysis of variance (ANOVA). Tukey’s test was used to verify the differences between the mean values. All calculations were conducted at a significance level of *P* ≤ 0.05. To illustrate the relationship between the influences of individual acids on *E. coli*, a hierarchical cluster analysis was used. The results were presented based on the tree grouping method taking the Euclidean distance and the Ward method as a measure. All statistical calculations were carried out using STATISTICA 13 PL (StatSoft, Poland) and Excel 2010 (Microsoft) computer programs.

## 3 Results and discussion

### 3.1 Antibacterial activity of benzoic acid derivatives (MIC and MBC)

MIC and MBC of acids differing in the number of groups, their types, and their positions at the benzoic ring were determined (Table 2). The pH values at which the MIC/MBC were determined were from 5.1 to 6.3/from 4.2 to 5.6 and did not inhibit the growth of *E. coli*. Attaching a hydroxyl or methoxyl substituent to benzoic acid weakens the antibacterial effect (except for the *ortho* position for the hydroxyl group). Two acids, Ba and 2hBa, showed the strongest antibacterial activity against *E. coli* O157 (MIC = 1 mg/mL). The least effective was 3,4,5hBa for which the MIC values were four times higher (MIC 4 mg/mL) than MIC for Ba. Other examined acids inhibited the development of *E. coli* at a concentration of 2 mg/mL. The location of the substituent in the *meta* and *para* position, in relation to the carboxyl group at the benzoic ring, and the presence of two substituents, caused a twofold weakening of bacteriostatic activity against *E. coli* in relation to Ba and 2hBa acids. No differences in bacteriostatic activity were found depending on the location as the number of methoxyl groups. Bactericidal values (MBC) of all acids were twice as high as MIC. Acid with hydroxyl substituent in *meta* position (3hBa) showed stronger bactericidal effects than acid with methoxyl substituent in the same position (3mBa).

**Table 2:** Minimal inhibitory concentrations (MICs) and minimal bactericidal concentration (MBCs) of phenolic acids against *E. coli*

Phenolic acids	*E. coli*	
	MIC (mg/mL) (pH)^a^	MBC (mg/mL) (pH)^a^
Benzoic acid (Ba)	1 (6.2)	2 (5.4)
2-Hydroxybenzoic acid (2hBa)	1 (6.3)	2 (5.6)
3-Hydroxybenzoic acid (3hBa)	2 (5.4)	4 (4.7)
4-Hydroxybenzoic acid (4hBa)	2 (5.5)	4 (4.9)
3,4-Dihydroxybenzoic acid (3,4hBa)	2 (5.6)	4 (4.9)
3,4,5-Trihydroxybenzoic acid (3,4,5hBa)	4 (5.1)	8 (4.4)
2-Methoxybenzoic acid (2mBa)	2 (5.5)	4 (4.8)
3-Methoxybenzoic acid (3mBa)	2 (5.4)	8 (4.2)
4-Methoxybenzoic acid (4mBa)	2 (5.5)	4 (5.2)
3,4-Dimethoxybenzoic acid (3,4mBa)	2 (5.1)	4 (5.6)

a The pH value of the medium.

The antibacterial properties of phenolic acids have already been tested by several researchers. Cueva et al. [13] found high antibacterial activity of benzoic acid, which inhibits the growth of *E. coli* O157:H7 at 1 mg/mL. Si et al. [14] determined the inhibition of *E. coli* O157:H7 growth by Ba, 2hBa, and 3hBa acids at a concentration of 0.5 mg/mL and the lack of antibacterial activity of 4hBa at the same concentration. Monte et al. [15] examined the effect of 2hBa acid on *E. coli* cells (MIC = 3.2 mg/mL; MBC = 5.0 mg/mL). In turn, Taguri et al. [16] verified the antibacterial properties of 3,4hBa acid in relation to *E. coli*, obtaining MIC = 2.6 mg/mL. In the study by Díaz-Gómez et al. [17], the MIC of 3,4,5hBa acid in relation to *E. coli* was determined at 3.25 mg/mL. Sanhueza et al. [18] for clinical isolates obtained very similar results of values of MIC for 3,4,5hBa acid for clinical isolates of *E. coli* 1.5–2.5 mg/mL and MIC 1.0–4.0 mg/mL. For 3,4hBa, 2,4hBa and 3,4hBa acids showed antibacterial activity at the concentration MIC = 1 mg/mL. For 4hBa and 3,4,5hBa acids, MIC > 1 mg/mL was determined [19].

The outer membrane of Gram-negative bacteria consists mainly of lipopolysaccharides (LPS), which reduce membrane permeability. The reduction in the penetration of lipophilic molecules such as phenolic acids is associated with an increased resistance of Gram-negative bacteria to antimicrobial compounds [20,21]. The value of the partition coefficient between octanol and water (log *P*) is an important parameter in determining the potential antimicrobial properties of new chemical compounds [22]. The high effectiveness of 2hBa acid may result not only from the high partition coefficient log *P* 2.3, which makes it easier to penetrate through cell membranes, but also from the low p*K*_a_ 2.94. The low p*K*_a_ value of 2hBa acid in relation to other acids (p*K*_a_ approximately 4.5) causes a rapid decrease in intracellular pH, which results in a short killing time at a low concentration in relation to the study by Yuan et al. [23] who demonstrated in their study that the substitution of −OH group in position 2 for Ba acid has a positive effect on the binding of 2hBa acid to blood serum. Conversely, the low effectiveness of 3,4,5hBa compared to other tested acids may result from the lowest log *P* 0.7 coefficient, which determines affinity to cell membranes. According to Oh and Jeon [4], the low susceptibility of *E. coli* to 3,4,5hBa is probably due to the presence of LPS, which is a critical barrier for this compound. In the conducted experiment, a high negative correlation (*r* = −0.82) was obtained between the antibacterial activity expressed as MIC and log *P* value. The higher the log *P* value, the stronger the inhibited growth of *E. coli* (lower MIC value).

### 3.2 Time kill study

The next stage of the study was to determine the time when *E. coli* cells were killed by the examined acids (Table 3). None of the examined acids in the MIC destroyed the cells for 24 h. Only three times higher values of MIC caused the death of the bacterial cells, which confirms the previously obtained MBC results. The pH values of the media varied little from 4.5 to 5.0, and hence, the pH had no effect on the difference of kill time of *E. coli* cells. The longest time of killing of *E. coli* cells in the concentration of 3MIC was found for Ba acid. The long kill time for Ba and 2hBa acids may be due to the lowest concentrations used (MIC = 1 mg/mL) which results in a higher pH of the environment. It is known that benzoic acid is more effective at low pH [2]. The mechanism action implies the diffusion through the membrane of the undissociated acid at low external pH. Later in the cytoplasm where pH is near neutrality, the weak acid would dissociate releasing one anion and one proton, with the subsequent acidification of the cytoplasm and accumulation of the anion [24]. The connection of both hydroxyl and methoxyl substituents to the benzoic acid significantly shortened the time of bacterial cells killing. 2hBa acid showed bactericidal effect after 240 min of culture, while 2mBa acid was characterized by four times shorter time (60 min). In turn, 4mBa acid had a killing time of 120 min, and its hydroxyl derivative (4hBa) needed half the time (60 min) to kill *E. coli*. There were no statistically significant differences in the bactericidal strength of acids differing in the location of the −OH group in *meta* and *para* positions and two acids which contained this group in both these positions. In the case of acids with the methoxyl group, no differences in bactericidal strength were shown if −OCH_3_ group was in *ortho* and *meta* positions and the acid containing two substituents in *meta* and *para* positions, and this suggests that the position of the methoxyl group in *meta* position is significant for bactericidal properties of these derivatives. This is confirmed in the study by Yuan et al. [23] who demonstrated that the introduction of the second group −OCH_3_ in position 3 into 4mBa acid reduces molecular polarization and increases the penetration capacity of the sites of phenol binding with blood albumin. Similarly, in the present study, 3,4mBa acid was characterized by a shorter killing time than 4mBa acid at the same MIC. Further research is needed to explain why most substituents reduce the kill time of *E. coli* cells.

**Table 3:** Time kill study

Phenolic acids	Time kill *E. coli*	
	MIC	3 MIC (pH)^a^
Benzoic acid (Ba)	—	24 h (4.9)
2-Hydroxybenzoic acid (2hBa)	—	240 min (5.0)
3-Hydroxybenzoic acid (3hBa)	—	60 min (4.5)
4-Hydroxybenzoic acid (4hBa)	—	60 min (4.6)
3,4-Dihydroxybenzoic acid (3,4hBa)	—	60 min (4.7)
3,4,5-Trihydroxybenzoic acid (3,4,5hBa)	—	60 min (4.5)
2-Methoxybenzoic acid (2mBa)	—	60 min (4.6)
3-Methoxybenzoic acid (3mBa)	—	60 min (4.5)
4-Methoxybenzoic acid (4mBa)	—	120 min (4.7)
3,4-Dimethoxybenzoic acid (3,4mBa)	—	60 min (4.8)

a The pH value of the medium.

### 3.3 *E. coli* biofilms

**Figure 1:**
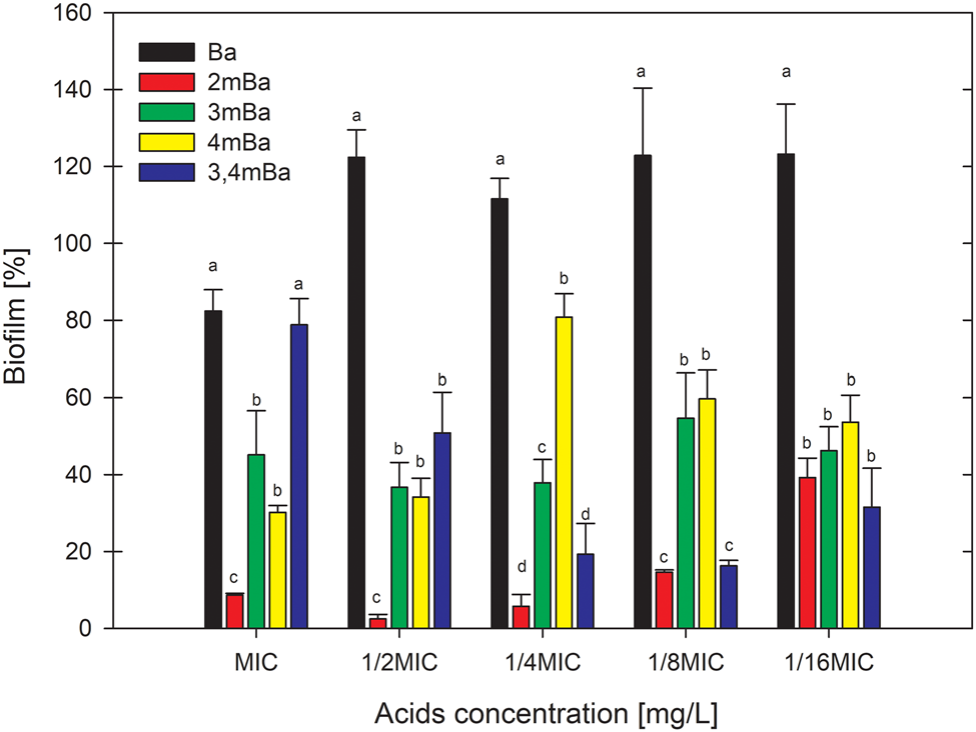
Graph with the effect of methoxyl derivatives of benzoic acid on the amount of biofilm formed by *E. coli.* Means with the same letter did not differ significantly.

**Figure 2:**
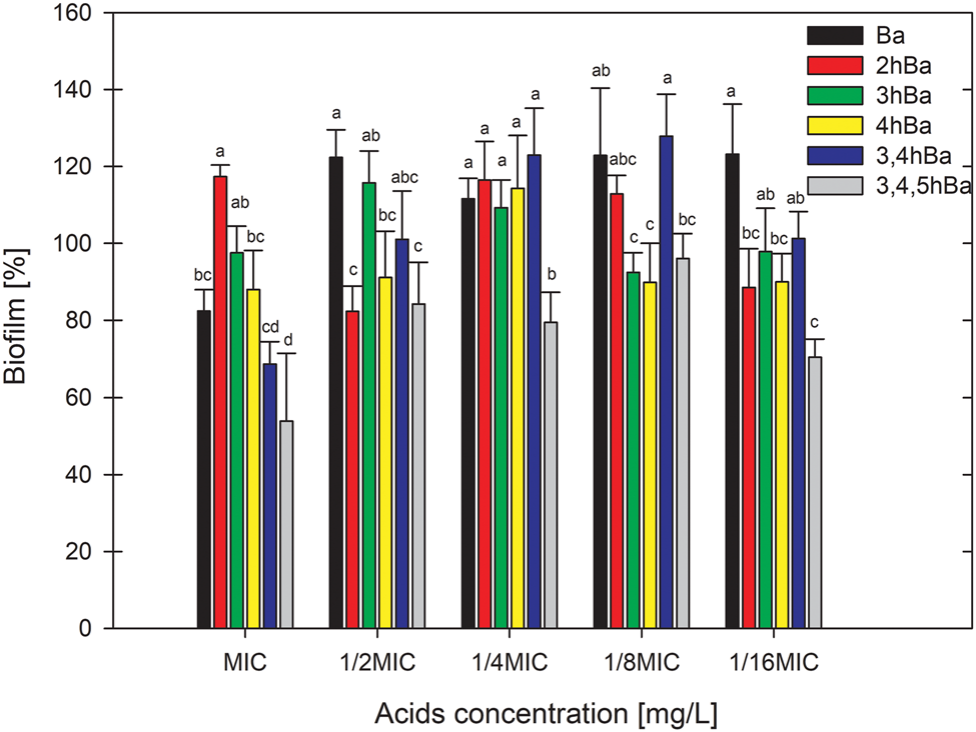
Graph with the effect of hydroxyl derivatives of benzoic acid on the amount of biofilm formed by *E. coli.* Means with the same letter did not differ significantly.

The effect of the number of groups, location of a single substituent, and concentration of hydroxyl and methoxyl derivatives of benzoic acid on the amount of biofilm produced in relation to the control sample containing no phenolic acid (100%) are presented in Figures 1 and 2. The benzoic acid, irrespective of its concentration, stimulated *E. coli* to form more biofilm than on a control medium containing no phenolic acid. The addition of 1, 2, or 3 hydroxyl groups had little effect on the amount of biofilm formed compared to benzoic acid. The location of a single −OH group in relation to the benzoic acid carboxylic group also affected the amount of biofilm formed by *E. coli*. It was found that the further the hydroxyl group is located in the acid, the less biofilm was formed.

The addition of 1 or 2 methoxyl groups to benzoic acid significantly reduced the amount of biofilm produced on average by 63%. The close location of the methoxyl group next to the carboxyl group in the acid resulted in a higher reduction of the biofilm in contrast to the hydroxyl group. The concentration of hydroxyl and methoxyl derivatives of benzoic acid had little effect on the amount of biofilm produced.

Several studies confirm the stimulating effect of antibacterial compounds on biofilm formation at low concentrations. This effect is a defensive response of bacteria to stressful environmental conditions. Stimulation of biofilm formation by low concentrations was described under the influence of antibiotics [25], alcohol [26], and plant extracts [27]. Borges et al. [28] in their study tested the influence of two phenolic acids, ferulic and gallic, on biofilm formation by *E. coli*. Both acids showed a 70% biofilm reduction compared to the control.

Comparing the action of the examined phenolic acids with benzoic acid, it can be stated that the presence and number of additional groups in the structure increase the properties against biofilm formation. Stronger biofilm inhibition properties of methoxybenzoic acids are most probably related to the increase in lipophilicity of the acids under the influence of the substituent with the methoxyl group, which is confirmed in the study by Sánchez-Maldonado et al. [29].

### 3.4 Hierarchical cluster analysis

**Figure 3:**
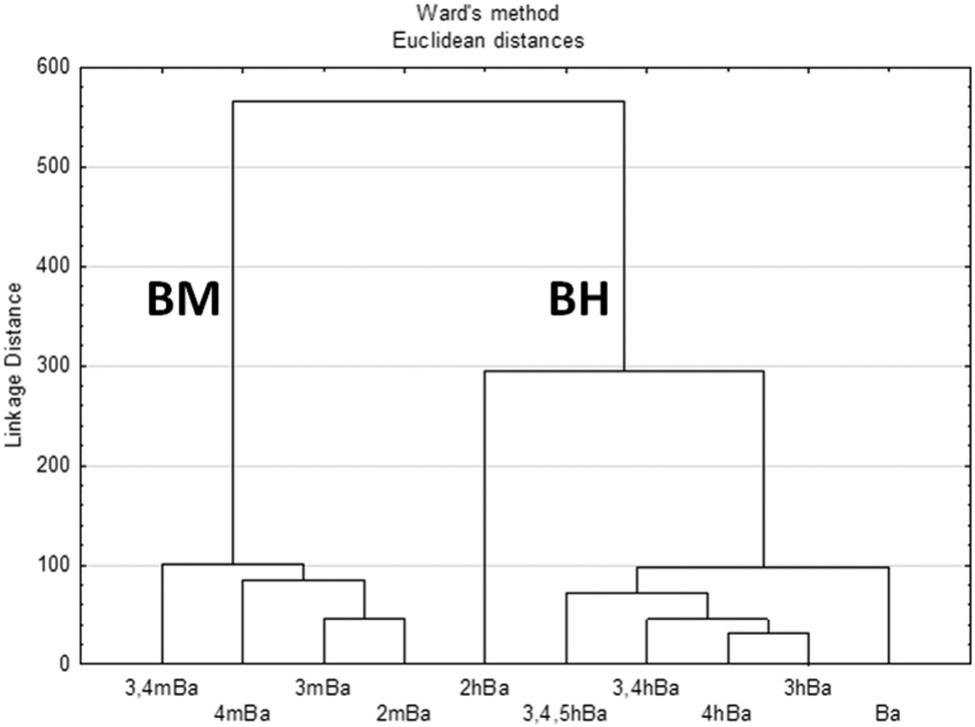
A dendogram showing the positional isomerism of phenolic acids for antimicrobial action using Ward’s minimum variance method.

The method of hierarchical cluster analysis was used to illustrate the effect of individual acids on *E. coli* (Figure 3). Two groups were separated, the first group BM included all derivatives of benzoic acid containing −OCH_3_ substituent, and the second group BH included all derivatives containing −OH substituent and benzoic acid. Subgroups with short Euclidean distances are visible (except 2hBa acid). The main factor determining the influence of the examined acids on the inhibition of *E. coli* growth is the type of substituent attached to the benzoic acid, followed by the number of groups present at the benzoic ring, and the least significant was the number of carbon atom at which a single substituent was present.

## 4 Conclusion

The most effective of the examined phenolic acids was 2hBa, characterized by the same bacteriostatic strength as Ba, but showing shorter time of *E. coli* cells killing. Derivatives containing methoxyl substituents are more active in limiting biofilm formation. It should be noted that the use of antibacterial compounds may provide prospects for reducing microbial hazards.

Systems of such compounds can be the basis for application in many industrial branches such as antibacterial preparations. Antibacterial substances can also be an integral component of various polymers used in the production of packaging (e.g., in the food industry). These substances can be added by coating the surface with the active ingredient as well as in the process of extruding polymers. However, we believe that further research should be continued in this scientific direction.
